# The antiarrhythmic drugs amiodarone and dronedarone inhibit intoxication of cells with pertussis toxin

**DOI:** 10.1007/s00210-024-03247-9

**Published:** 2024-07-03

**Authors:** Jinfang Jia, Stefanie Lietz, Holger Barth, Katharina Ernst

**Affiliations:** 1 Institute of Experimental and Clinical Pharmacology, Toxicology and Pharmacology of Natural Products, Ulm University Medical Center, Ulm, Germany; 2 Present Address: Department of Respiratory Medicine, Shanghai Sixth People’s Hospital Affiliated to Shanghai Jiao Tong University School of Medicine, 600 Yishan Road, Shanghai, 200233 China

**Keywords:** Amiodarone, Dronedarone, Pertussis toxin, Whooping cough, Toxin inhibitor, Drug repurposing

## Abstract

Pertussis toxin (PT) is a virulent factor produced by *Bordetella pertussis*, the causative agent of whooping cough. PT exerts its pathogenic effects by ADP-ribosylating heterotrimeric G proteins, disrupting cellular signaling pathways. Here, we investigate the potential of two antiarrhythmic drugs, amiodarone and dronedarone, in mitigating PT-induced cellular intoxication. After binding to cells, PT is endocytosed, transported from the Golgi to the endoplasmic reticulum where the enzyme subunit PTS1 is released from the transport subunit of PT. PTS1 is translocated into the cytosol where it ADP-ribosylates inhibitory α-subunit of G-protein coupled receptors (Gαi). We showed that amiodarone and dronedarone protected CHO cells and human A549 cells from PT-intoxication by analyzing the ADP-ribosylation status of Gαi. Amiodarone had no effect on PT binding to cells or in vitro enzyme activity of PTS1 but reduced the signal of PTS1 in the cell suggesting that amiodarone interferes with intracellular transport of PTS1. Moreover, dronedarone mitigated the PT-mediated effect on cAMP signaling in a cell-based bioassay. Taken together, our findings underscore the inhibitory effects of amiodarone and dronedarone on PT-induced cellular intoxication, providing valuable insights into drug repurposing for infectious disease management.

## Introduction

Pertussis, also known as whooping cough, is caused by *Bordetella pertussis* and remains a persistent global health concern despite vaccination efforts (Kilgore et al. 2016; Yeung et al. 2017). At the heart of its pathogenesis lies pertussis toxin (PT), a multifaceted virulence factor central to the bacterium’s ability to cause disease (Pittman 1984; Locht et al. 2011; Scanlon et al. 2019).

Comprising an A-protomer PTS1 coupled with a pentameric B-subunit, PT forms a holo-toxin adept at binding to cell surfaces through sialoglycoproteins, facilitating its internalization via endocytosis (Tamura et al. 1982; Armstrong et al. 1988; Witvliet et al. 1989; Hausman and Burns 1993; Weiss et al. 1993; Stein et al. 1994). Once internalized, PT embarks on a retrograde voyage through the Golgi apparatus to the endoplasmic reticulum (ER) (el Bayâ et al. 1997; Plaut and Carbonetti 2008). It is within the ER that the toxin undergoes critical disassembly, triggered by ATP binding, resulting in the dissociation of PTS1 from the holotoxin (Burns and Manclark 1986; Hazes et al. 1996; Pande et al. 2006; Plaut et al. 2016; Banerjee et al. 2016). This liberated PTS1 becomes a target for ER-associated degradation (ERAD) machinery, translocating it from the ER to the cytosol (Hazes and Read 1997). Host cell chaperones and enzymes actively participate in this process, highlighting the complex interplay between pathogen and host (Ernst et al. 2018, 2021; Kellner et al. 2019, 2021; Ernst 2022). Lacking crucial lysine residues, PTS1 escapes the subsequent proteasomal degradation (Worthington and Carbonetti 2007).

Upon entering the cytosol, PTS1 executes its cytotoxic action by covalently transferring an ADP-ribose moiety from NAD^+^ onto the α-subunit of inhibitory G-proteins (Gαi) associated with G-protein-coupled receptors (GPCRs) (Katada and Ui 1982; Bokoch et al. 1983; Sakari et al. 2022). This enzymatic modification disrupts crucial cellular signaling pathways, specifically the cAMP signaling cascade, exerting diverse effects depending on the cell type affected (Carbonetti 2015). The consequences of PT’s interference with cellular signaling pathways are profound. It manifests as stimulation of insulin secretion, inhibition of immune cell recruitment to the lungs resulting in reduced inflammation, and disturbances in lymphocyte trafficking and adhesion molecule expression, thereby impacting immune responses (Carbonetti 2015).

Clinically, pertussis manifests in severe, persistent coughing, often leading to secondary complications such as vomiting, pneumothorax, pneumonia, seizures, and life-threatening conditions, especially in infants (Mattoo and Cherry 2005; Paddock et al. 2008). Despite widespread vaccination, pertussis cases have resurged globally, inflicting substantial morbidity and mortality, particularly among children under five years old (Althouse and Scarpino 2015; Esposito et al. 2019; Locht and Antoine 2021).Addressing pertussis effectively remains a challenge, especially concerning late-stage administration of antibiotics, which fail to alleviate symptoms caused by PT’s virulence. Consequently, PT emerges as a promising therapeutic target for developing novel strategies against pertussis (Carbonetti 2016; Kilgore et al. 2016; Ernst 2022).

This study aims to evaluate antiarrhythmic drugs amiodarone and dronedarone as potential inhibitors of PT, exploring their promise as novel therapeutic strategies against whooping cough. Amiodarone and dronedarone are antiarrhythmic medications used to manage irregular heart rhythms (Singh and Vaughan Williams 1970; Goldschlager et al. 2007). Amiodarone is highly effective but associated with potential side effects affecting various organs, including the lungs, liver, thyroid, and eyes (Raeder et al. 1985). In contrast, dronedarone was developed with the aim of reducing adverse effects, particularly on organs like the lungs and thyroid, while still addressing irregular heartbeats (Yalta et al. 2009; Khan et al. 2017). Both drugs function by altering the heart’s electrical activity to restore and maintain a normal heartbeat (Kodama et al. 1997; Ghovanloo et al. 2016).

Recently, it was shown that amiodarone protects against cell intoxication caused by *Clostridioides difficile* toxins TcdB and TcdA (Schumacher et al. 2023). An inhibitory effect was also reported on *Bacillus anthracis* anthrax toxin (Sanchez et al. 2007). This prompted further investigation into whether amiodarone and dronedarone possess similar inhibitory properties against PT. In this study, we aim to explore the potential inhibitory effects of amiodarone on PT and elucidate whether it could serve as a protective agent against PT-induced cellular intoxication. This investigation seeks to expand our understanding of amiodarone’s broader inhibitory capabilities against bacterial toxins, potentially uncovering new avenues for therapeutic interventions against pertussis.

## Materials and methods

### Protein expression and purification

Following the previously described methods (Ashok et al. 2020), recombinant proteins, namely PTS1 and Gαi, were expressed and purified. His-tagged PTS1 and Gαi were expressed in BL21(DE3) and then purified through a two-step process involving HisTrap HP columns followed by size exclusion chromatography.

### Cell culture

Chinese hamster ovary cells strain K1 (CHO-K1) were acquired from DSMZ (Leibniz Institute DSMZ-German Collection of Microorganisms and Cell Cultures GmbH) and cultured in a blend of DMEM and HAM’s F12 supplemented with 5% heat-inactivated fetal calf serum, 1 mM sodium pyruvate, and penicillin–streptomycin (1:100). The cells were maintained at 37 °C with 5% CO_2_. A549 human lung adenocarcinoma cells were sourced from ATCC and cultivated at 37 °C with 5% CO_2_ in DMEM supplemented with 10% FCS, 1 mM sodium pyruvate, 0.1 mM non-essential amino acids, 100 U/mL of penicillin, and 100 μg/mL of streptomycin. Cells were trypsinized and transferred to a 10 cm culture dish every two to three days for a maximum of 25 cycles.

### Sequential ADP-ribosylation of Gαi in lysates from toxin-treated cells

Cells were first subjected to pre-incubation with specific inhibitors at 37 °C and then treated with PT (10 ng/mL, Merck Sigma) for designated periods. After treatment, the cells underwent triple washing with PBS and were subsequently frozen at − 20 °C overnight to facilitate cell lysis. Cell lysates in 30 μL of ADP-ribosylation buffer (0.1 mM Tris–HCl (pH 7.6), 20 mM DTT, and 0.1 μM ATP, complete protease inhibitor (Roche)) were incubated at room temperature for 40 min with 100 nM PTS1 and biotin-labeled NAD^+^ (8.3 μM; R&D Systems, Minneapolis, MA, USA) to enable in vitro ADP-ribosylation of Gαi, that was not modified before in the living cells. Post-incubation, the samples were subjected to SDS-PAGE, followed by blotting. Detection of biotin-labeled (i.e., ADP-ribosylated) Gαi was carried out using streptavidin-peroxidase (Strep-POD, Sigma-Aldrich, Merck, St. Louis, MO, USA) with the enhanced chemiluminescence (ECL) system. To ensure consistent protein amounts, Ponceau S staining and Hsp90 detection with a specific antibody (Santa Cruz, Dallas, TX, USA) were performed. Densitometric quantification of Western blot signals was conducted using the ImageJ histogram tool (v1.53 k, National Institute of Health, Bethesda, MD, USA), with values normalized based on the amount of loaded protein.

### In vitro enzyme activity assay

Recombinant Gαi (0.8416 μM) was subjected to a 30 min incubation at room temperature with indicated inhibitors. As a control, Gαi was also incubated with DMSO, functioning as the solvent for the inhibitors. The final concentration of DMSO was compared to the highest DMSO concentration used in the inhibitor experiments. 100 nM PTS1 and 10 μM biotin-labeled NAD^+^ were introduced and allowed to incubate for an additional 30 min at room temperature. The samples were further processed as described above.

### Detection of PT binding to cells

Cells were initially exposed to the respective inhibitors for 30 min at 37 °C, followed by a 15-min incubation on ice. Subsequently, PT was introduced and allowed to incubate for 40 min on ice. Following this, the cells underwent two washes with PBS, and Laemmli buffer containing DTT was added. The samples were then analyzed using SDS-PAGE and Western blotting, employing a specific antibody (Santa Cruz, #sc-57639 (63.1G9)) for PTS1 detection. Signal intensity was quantified via densitometry using ImageJ, and the confirmation of uniform loading was ensured through Hsp90 staining.

### Immunolabeling and fluorescence microscopy

CHO cells were incubated with the respective inhibitors in ibidi 8-well µ-plates for 30 min. Then, cells were incubated with PT for 4 h. Afterward, the cells underwent three cold PBS washes and were fixed with 4% PFA for 20 min at room temperature (RT). Following another three cold PBS washes, permeabilization was performed with Triton X-100 (0.4% in PBS) for 5 min at RT, followed by three additional PBS washes. Autofluorescence was quenched with 100 nM glycine in PBS for 2 min at RT. Cells were subjected to blocking with 10% normal goat serum (Jackson ImmunoResearch, Philadelphia, PA, USA) and incubated with an anti-PTS1 antibody (diluted 1:200 in the blocking solution) for 1 h at 37 °C. After washing with PBST, the cells were probed with a fluorescence-labeled secondary antibody, anti-mouse488 (diluted 1:1000 in the blocking solution; Invitrogen, Waltham, MA, USA), and subjected to Hoechst staining (diluted 1:10,000 in PBST) for 5 min. Following five additional washes, imaging was performed using the Keyence BZ-X810 fluorescence microscope (Osaka, Japan) with a Plan Apochromat 40X objective and BZ-X filters for DAPI (OP-87762) and GFP (OP-87763).

### iGIST bioassay

The iGIST bioassay was performed as described before (Paramonov et al. 2020; Kling et al. 2021; Jia et al. 2024). In brief, the assay is based on genetically modified HEK293 cells expressing Gαi-coupled somatostatin receptor 2 (SSTR2) GPCR and the luminescent cAMP probe GloSensor-22F. Cells were treated with inhibitors for 1 h, followed by PT (100 ng/mL) or a matched buffer for 4 h. Afterward, cells were exposed to an inducing medium containing the GloSensor reagent (Promega) and the phosphodiesterase inhibitor IBMX (Merck Sigma). Luminescence reflecting cAMP levels was recorded using the Orion microplate luminometer. Forskolin (activator of adenylate cyclase, Merck Sigma) and octreotide acetate (activator of SSTR2, Bachem, Bubendorf, Switzerland) were added to stimulate cells, and the area under the curve (AUC) was calculated for kinetic analysis using GraphPad Prism software.

### Reproducibility of experiments and statistics

Each experiment was independently conducted at least three times, with the number of replicates specified in the corresponding figures. The figures display representative results, and for presentation purposes, Western blots were cropped. Statistical analysis, as outlined in the figure legends, was carried out using the GraphPad Prism software. Significance levels are indicated as follows: ****p < 0.0001, ***p < 0.001, **p < 0.01, *p < 0.05, and ns = not significant (*p* > 0.05).

## Results

### Amiodarone reduces levels of ADP-ribosylated Gαi in PT-treated cells

**Fig. 1:**
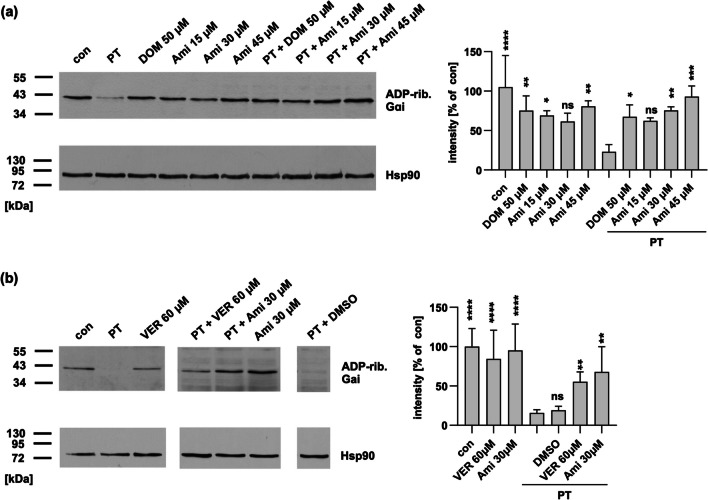
Amiodarone treatment results in reduced ADP-ribosylated Gαi levels. CHO cells (**a**) or A549 cells (**b**) were pre-incubated with indicated concentrations of amiodarone (Ami) for 1 h. For control, cells were left untreated (con) or treated with the established inhibitor domperidone (DOM) or VER. Then, PT (10 ng/ml (**a**), 50 ng/ml (**b**)) was added to indicated samples. After 4 h of incubation, cells were lysed, and lysates incubated with PTS1 and biotin-labeled NAD^+^. SDS-PAGE and Western blotting allowed detection of ADP-ribosylated, biotin-labeled Gαi. Hsp90 was detected as loading control. Signals were quantified and normalized to Hsp90 signals as well as to untreated controls. Values are given as mean ± SEM (*n* = *n* ≥ 4 from 4 independent experiments). Significance was tested against samples treated only with PT using one-way ANOVA with Dunnett’s multiple comparisons test. ****p < 0.0001, ***p < 0.001, **p < 0.01, *p < 0.05, ns = not significant. Images were cropped for display purposes only

Once PTS1 reaches the cytosol of target cells, it ADP-ribosylates its specific substrate Gαi. This can be analyzed via Western blotting by lysing the cells and subsequently incubating the lysates with fresh PTS1 in the presence of a biotin-labeled co-substrate, NAD^+^. During this incubation, PTS1 transfers the ADP-ribose moiety and with that the biotin label onto Gαi, thereby labeling Gαi that has not been previously modified in the cells. Biotin-labeled, i.e., ADP-ribosylated Gαi, was then detected in Western blot analysis using streptavidin. The ADP-ribosylation status of Gαi was analyzed in Chinese hamster ovary (CHO) cells (Fig. 1a), which are often used to analyze PT intoxication as well as in human adenocarcinomic alveolar basal epithelial cells, which are pathophysiologically more relevant cell line (Fig. 1b). A strong signal in this assay suggests no intoxication, as it indicates that the entire pool of Gαi in the cell can undergo ADP-ribosylation during subsequent in vitro incubation with biotin-labeled NAD^+^. Samples from cells treated with PT exhibit a weak signal (Fig. 1). However, samples treated with amiodarone prior to PT treatment show a signal comparable to untreated control samples, indicating protection from PT intoxication by amiodarone (Fig. 1). As a control, domperidone (DOM) or VER-155008 (VER), inhibitors of Hsp70, were used, as previous studies have demonstrated their protective effect against PT intoxication. Notably, amiodarone alone had only a minor impact on the detection of the ADP-ribosylation status of Gαi.

### Amiodarone has no effect on enzyme activity in vitro or cell binding of PT

**Fig. 2:**
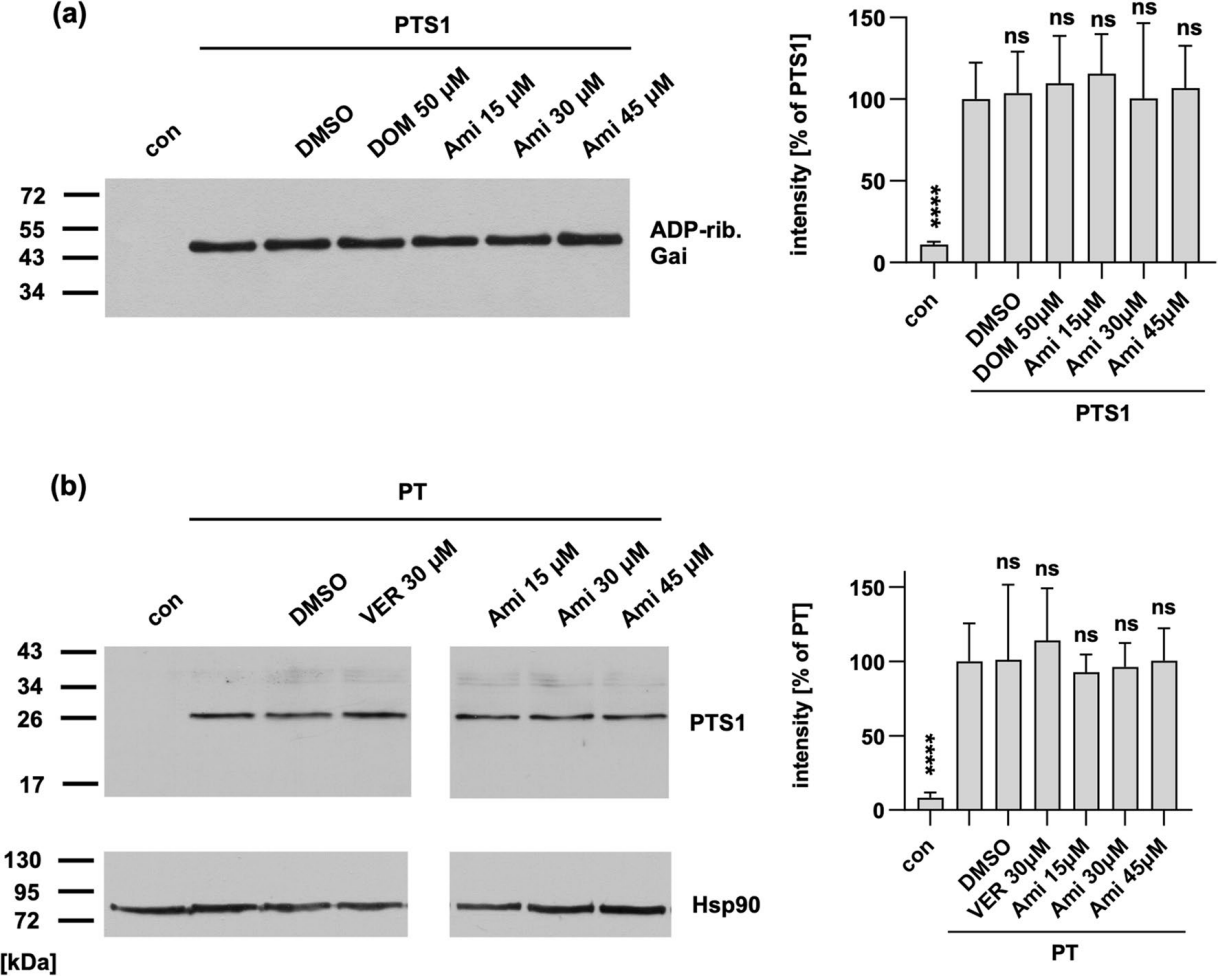
Amiodarone had no effect on enzyme activity or cell binding of PT. **a** Recombinant Gαi was incubated with 100 nM PTS1 and biotin-labeled NAD^+^ for 30 min at room temperature in the presence or absence of indicated inhibitors. For control, solvent of amiodarone (Ami), DMSO, was tested or PTS1 was not added to the sample (con). ADP-ribosylated, biotin-labeled Gαi was detected by Western blotting. Signals were quantified and values are displayed as mean ± SEM (*n* ≥ 6 from 6 independent experiments). Significance was tested against samples treated only with PTS1 using one-way ANOVA with Dunnett’s multiple comparisons test. **b** CHO cells were pre-incubated with indicated inhibitors, DMSO as solvent control or left untreated for 30 min at 37 °C. Then, cells were incubated on ice for 10 min. PT (500 ng/ml) was added and incubation was continued on ice for 30 min. After washing, samples were subjected to SDS-PAGE and Western blotting. PTS1 was detected by a specific antibody. Hsp90 was detected to confirm equal loading. Signals were quantified, normalized to Hsp90 signal and samples treated only with PT. Values are displayed as mean ± SEM (*n* ≥ 4 from 4 independent experiments). Significance was tested against samples treated only with PT using one-way ANOVA with Dunnett’s multiple comparisons test. ****p < 0.0001, ns = not significant. Cropping of images was done solely for display purposes

Next, the objective was to elucidate how amiodarone disrupts PT activity, thereby shielding cells from intoxication. Therefore, we incubated the recombinant enzyme subunit PTS1 with or without amiodarone, alongside recombinant Gαi and biotin-labeled NAD^+^. This facilitated ADP-ribosylation and therefore biotin-labeling of Gαi, detectable via Western blotting using streptavidin to visualize the biotin-label. As controls, samples were treated with DMSO, the solvent of amiodarone, or with DOM. The results depicted in Fig. 2a reveal equal signal intensities irrespective of amiodarone treatment, indicating that amiodarone did not affect in vitro PTS1 activity.

Another aspect of the intoxication process potentially affected by amiodarone is the interaction of PT with cells. To evaluate whether amiodarone influences PT binding to the cell surface, experiments were conducted at 4 °C, a temperature conducive to PT binding but not its internalization via endocytosis. The binding of PT to cells was subsequently assessed from cell lysates using a specific antibody targeting PTS1 in Western blot analysis. It was observed that amiodarone did not impede PT’s binding to cells, as depicted in Fig. 2b.

### PTS1 signal is reduced in cells treated with amiodarone

**Fig. 3:**
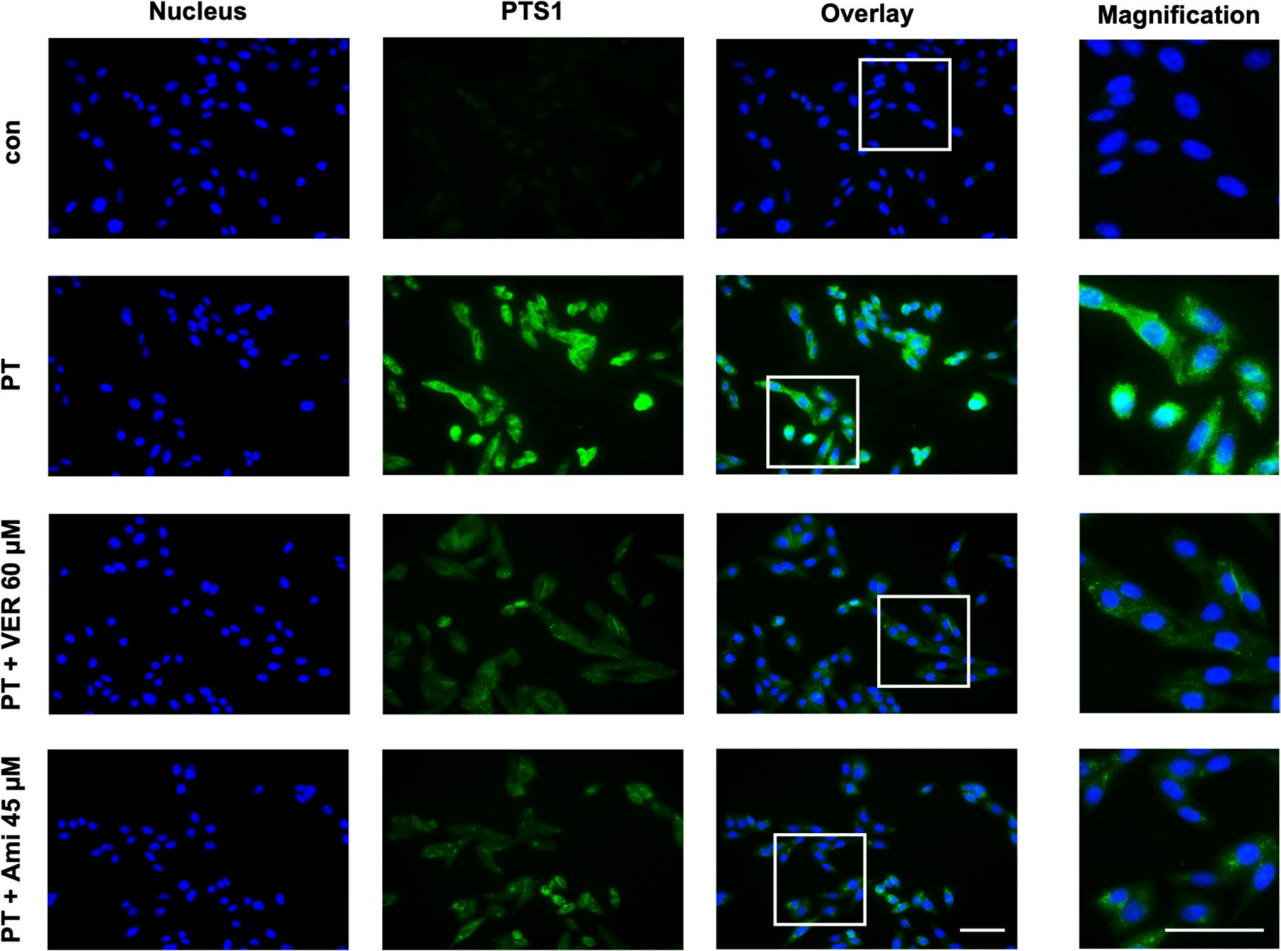
When cells were exposed to amiodarone (Ami), a reduction in the PTS1 signal is evident within the cells. CHO cells underwent a 30 min pre-incubation with Ami or VER at 37 °C, while untreated cells served as the control. Subsequently, PT (100 ng/mL) was administered for a 4 h period. After washing, cells were fixed and permeabilized, followed by detection of PTS1. Nuclei were stained using Hoechst dye, and images were captured randomly using a Keyence fluorescence microscope. Magnified areas are indicated by white squares. The scale bar is 50 µm

Having established that amiodarone does not impede PT binding to cells or enzyme activity in vitro, our focus shifted to investigating whether amiodarone affects the trafficking of PTS1 into the cytosol. To do so, we utilized a specific antibody targeting PTS1, known for its ability to recognize dissociated PTS1 from the B-subunit pentamer, indicative of primarily cytosolic PTS1 localization (Ernst et al. 2018, 2021). The fluorescence signals emitted by PTS1 were notably reduced in samples treated with amiodarone or VER (for control) compared to those treated solely with PT (Fig. 3). These results suggest that amiodarone inhibits the translocation of PTS1 into the cytosol of cells.

### Dronedarone inhibits intoxication of cells with PT in a concentration-dependent manner

**Fig. 4:**
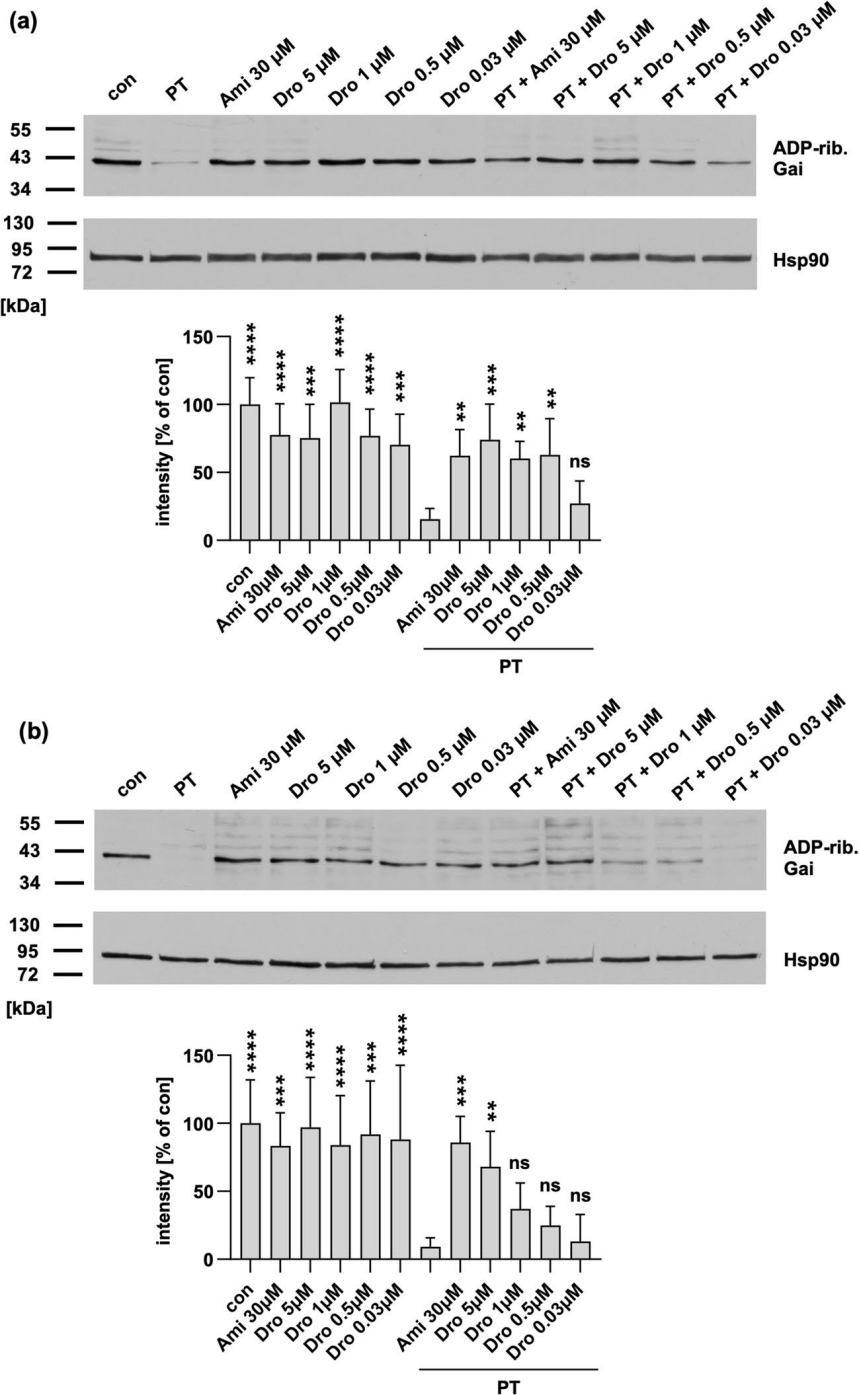
Dronedarone treatment leads to a reduction in the levels of ADP-ribosylated Gαi in a concentration-dependent manner. CHO cells (**a**) or A549 cells (**b**) underwent a 1 h pre-incubation with varying concentrations of dronedarone (Dro). For control purposes, cells were either left untreated (con) or treated with amiodarone (Ami) in comparison. Following this, PT (10 ng/ml (**a**), 50 ng/ml (**b**)) was introduced to the designated samples. After a 4 h incubation period, cell lysates were prepared and incubated with PTS1 and biotin-labeled NAD^+^. Detection of ADP-ribosylated, biotin-labeled Gαi was accomplished through SDS-PAGE and Western blotting, with Hsp90 serving as the loading control. Signal quantification was performed, and values were normalized to Hsp90 signals and untreated controls. The values represent mean ± SEM (*n* ≥ 4 from 4 independent experiments in **a**, *n* ≥ 5 from 5 independent experiments in **b**). Significance was determined by comparison to samples treated exclusively with PT using one-way ANOVA with Dunnett’s multiple comparisons test. ****p < 0.0001, ***p < 0.001, **p < 0.01, *p < 0.05, ns = not significant

Dronedarone, a derivative of amiodarone, lacks the iodine moiety present in amiodarone and was developed to reduce side effects for example associated with iodine, such as thyroid dysfunction. To evaluate its efficacy, we tested dronedarone’s impact on cell intoxication with PT in CHO and A549 cells (Fig. 4). In CHO cells, concentrations ranging from 0.5 to 5 µM significantly inhibited PT intoxication without markedly affecting the detection of ADP-ribosylation of Gαi on their own (Fig. 4a). In A549 cells, significant inhibition was observed at concentrations of 5 µM. Notably, compared to amiodarone, lower concentrations of dronedarone exhibited a protective effect.

### Dronedarone attenuates the effect of PT on cAMP signaling

In the cytosol, PTS1-mediated ADP-ribosylation disrupts Gαi’s ability to bind to GPCRs, rendering it ineffective in inhibiting adenylate cyclase activity, thus compromising the efficacy of agonist stimulation of inhibitory GPCRs (Katada and Ui 1982; Bokoch et al. 1983). The iGIST bioassay was established to evaluate PT’s impact on cAMP signaling in living cells (Ashok et al. 2020). HEK293 cells overexpressing the Gαi-coupled GPCR somatostatin receptor 2 (SSTR2), which is inducible with octreotide, along with a luminescent cAMP probe are used. These cells were treated with forskolin to stimulate adenylate cyclase activity and with octreotide to activate SSTR2, resulting in decreased cAMP levels due to Gαi-mediated inhibition of adenylate cyclase, a process that PT can reverse.

**Fig. 5:**
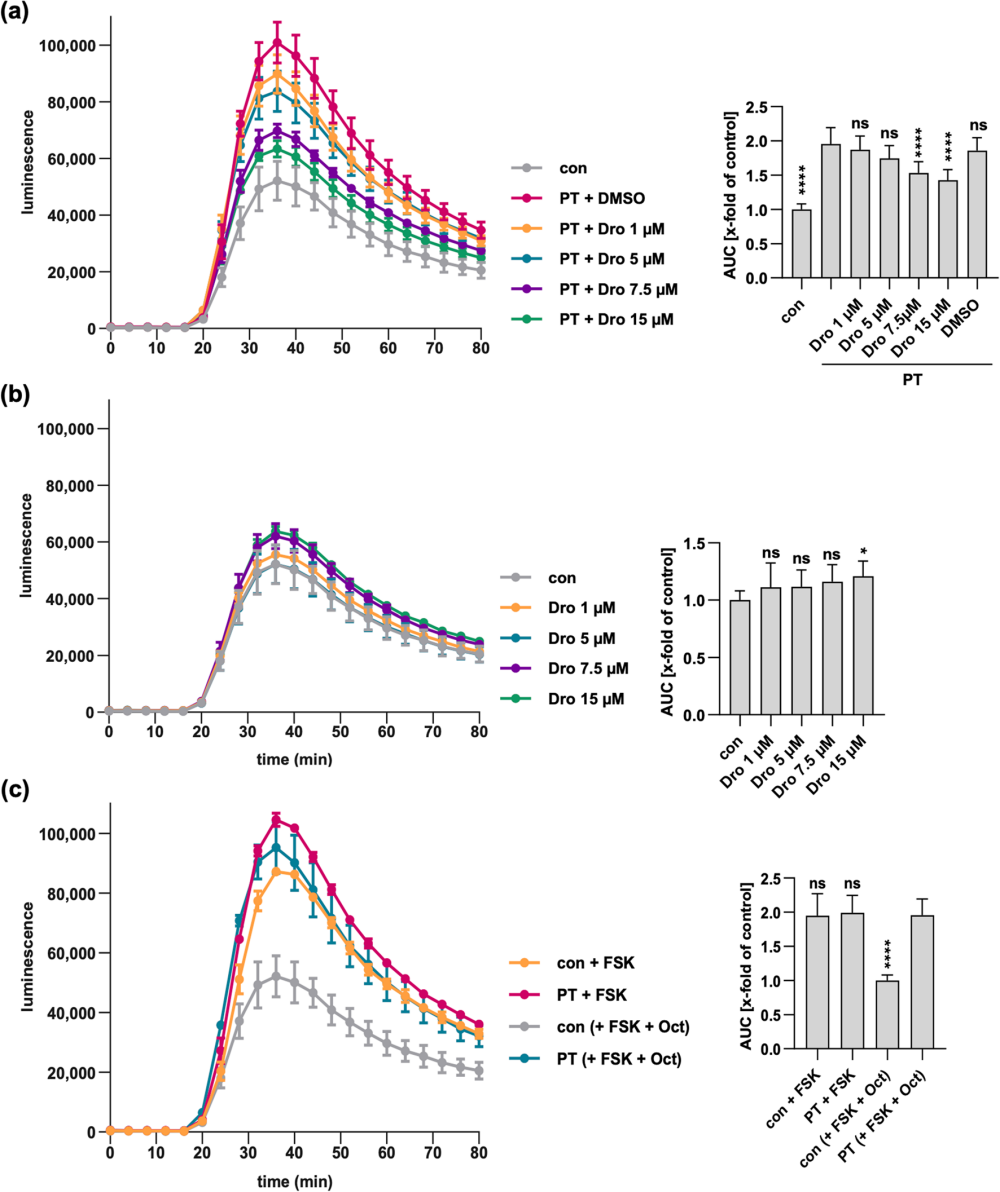
Dronedarone attenuates the effect of PT on cAMP signaling. Cells were exposed to various concentrations of Dronedarone (Dro) or DMSO (equivalent to 15 µM Dro) for 1 h followed by 100 ng/ml of PT for a 4 h duration at 37 °C (**a**) or left untreated as a control (**b**). Additionally, a control group received neither inhibitor nor PT (con). Subsequently, inducing medium containing a luciferase substrate for the luminescent cAMP biosensor was added. After a 15 min baseline measurement, forskolin (FSK), an adenylate cyclase activator, and octreotide acetate (Oct), an activator of SSTR2 were added. Luminescence data were recorded over an 80 min period. cAMP kinetic curves from a single representative experiment are displayed on the left. The values present mean ± SD, derived from three samples within a single experiment. Bar graphs on the right depict the baseline-subtracted area under the curve (AUC) obtained from at least three separate experiments, expressed as a percentage relative to control samples. The mean ± SEM values, obtained from at least six values of three independent experiments, are provided. For control purposes, cells were treated with FSK only or PT in combination with FSK, excluding Oct to observe maximal cAMP response. The values for control samples and samples treated with PT, FSK, and Oct mirror those in panel (**a**). Statistical analysis involved a mixed-effects assessment along with Dunnett’s multiple comparisons test. The presented values correspond to samples treated exclusively with PT (**a**, **c**) or the control (**b**). ****p < 0.0001, *p < 0.05, ns = not significant

Amiodarone was tested in this assay. However, it caused damage to the HEK293 cells, rendering it impossible to assess its impact on cAMP signaling. In contrast, dronedarone only marginally affected cAMP levels on its own (Fig. 5b) and showed a reduction in cAMP signals in a concentration dependent manner compared to cells treated only with PT (Fig. 5a). Peak cAMP levels were observed when cells were solely treated with forskolin or when exposed to PT in conjunction with forskolin and octreotide (Fig. 5c). These findings indicate that dronedarone can mitigate PT-induced alterations in host cell cAMP signaling.

In summary, our findings demonstrate that amiodarone provides protection against PT intoxication in both CHO and human A549 cells. While amiodarone did not hinder PT binding to cells or PTS1 enzyme activity in vitro, it did lead to diminished signals of PTS1 within the cells, indicating interference with intracellular trafficking. Furthermore, the amiodarone derivative, dronedarone, exhibited similar protective effects against PT intoxication in CHO and A549 cells and mitigated PT-mediated effects on cAMP signaling.

## Discussion

PT is a crucial virulence factor produced by *Bordetella pertussis*, the causative agent of whooping cough. PT exerts its pathogenic effects by ADP-ribosylating heterotrimeric G proteins, disrupting cellular signaling pathways. Here, we explored the potential of two antiarrhythmic drugs, amiodarone and dronedarone, in mitigating PT-induced cellular intoxication. Through a series of experiments, we demonstrate that both amiodarone and dronedarone effectively inhibit PT-intoxication of cells, offering insights into their potential therapeutic applications beyond arrhythmia management.

Previous studies have revealed amiodarone’s ability to inhibit other bacterial AB-type toxins, including *Bacillus anthracis* lethal toxin (LT) and edema toxin (ET) as well as *Corynebacterium diphtheriae* toxin (DT) (Sanchez et al. 2007), while not affecting *Vibrio cholerae* toxin (CT) and shiga toxin (Sanchez et al. 2007; Piccoli et al. 2011) (Table 1). This differential inhibition was attributed to amiodarone’s interference with the acidification of endosomes necessary for the translocation of short-trip toxins such as LT, ET, and DT, contrasting with long-trip toxins that traverse a different intracellular route like CT and shiga toxin (Sanchez et al. 2007; Piccoli et al. 2011).

**Table 1:** Effect of amiodarone/dronedarone on bacterial toxins

Toxin	Inhibited by amiodarone	Inhibited by dronedarone	Proposed inhibition mechanism
*B. anthracis* lethal toxin (LT) (Sanchez et al. 2007)	Yes	Not tested	Interference with endosomal acidification required for toxin translocation to the cytosol
*B. anthracis* edema toxin (ET) (Sanchez et al. 2007)	Yes	Not tested	
Diphtheria toxin (DT) (Sanchez et al. 2007)	Yes	Not tested	
Cholera toxin (CT) (Sanchez et al. 2007)	No	Not tested	
Shiga toxin (Piccoli et al. 2011)	No	Not tested	
*C. difficile* TcdA, TcdB (Schumacher et al. 2023) (Matylitsky et al. 2024, under revision at Naunyn Schmiedeberg’s Archives of Pharmacology)	Yes	Yes	Interference with translocation pore of the toxin
Pertussis toxin (PT) (this study)	Yes	Yes	Interference of intracellular toxin trafficking

Recently, it was discovered that *Clostridioides difficile* toxins TcdA and TcdB are also inhibited by amiodarone (Schumacher et al. 2023). The rationale behind this study was amiodarone’s inhibition on cholesterol biosynthesis (Allen et al. 2020; Simonen et al. 2020; Barsi et al. 2022) because it was shown earlier that uptake of TcdA and TcdB depends on membrane cholesterol (Giesemann et al. 2006; Papatheodorou et al. 2019, 2021). However, the study revealed an additional mode of inhibition. Amiodarone protected cells and human intestinal organoids from TcdA/TcdB intoxication most likely by interference with the translocation pore (Schumacher et al. 2023). As a multichannel inhibitor, blocking potassium channels, it seems reasonable that amiodarone might act as a pore blocker also for pores formed by toxin subunits (Singh and Vaughan Williams 1970; Kodama et al. 1997; Nattel and Singh 1999; Wu et al. 2008; Ghovanloo et al. 2016).

Results from these studies, including our own, were primarily obtained using cell lines and in vitro experimentation. While these findings are promising, they may not fully replicate the complex interactions and responses that occur in a living organism. Further in vivo studies are necessary to confirm the efficacy and safety of amiodarone and dronedarone in mitigating toxin-induced effects.

**Fig. 6:**
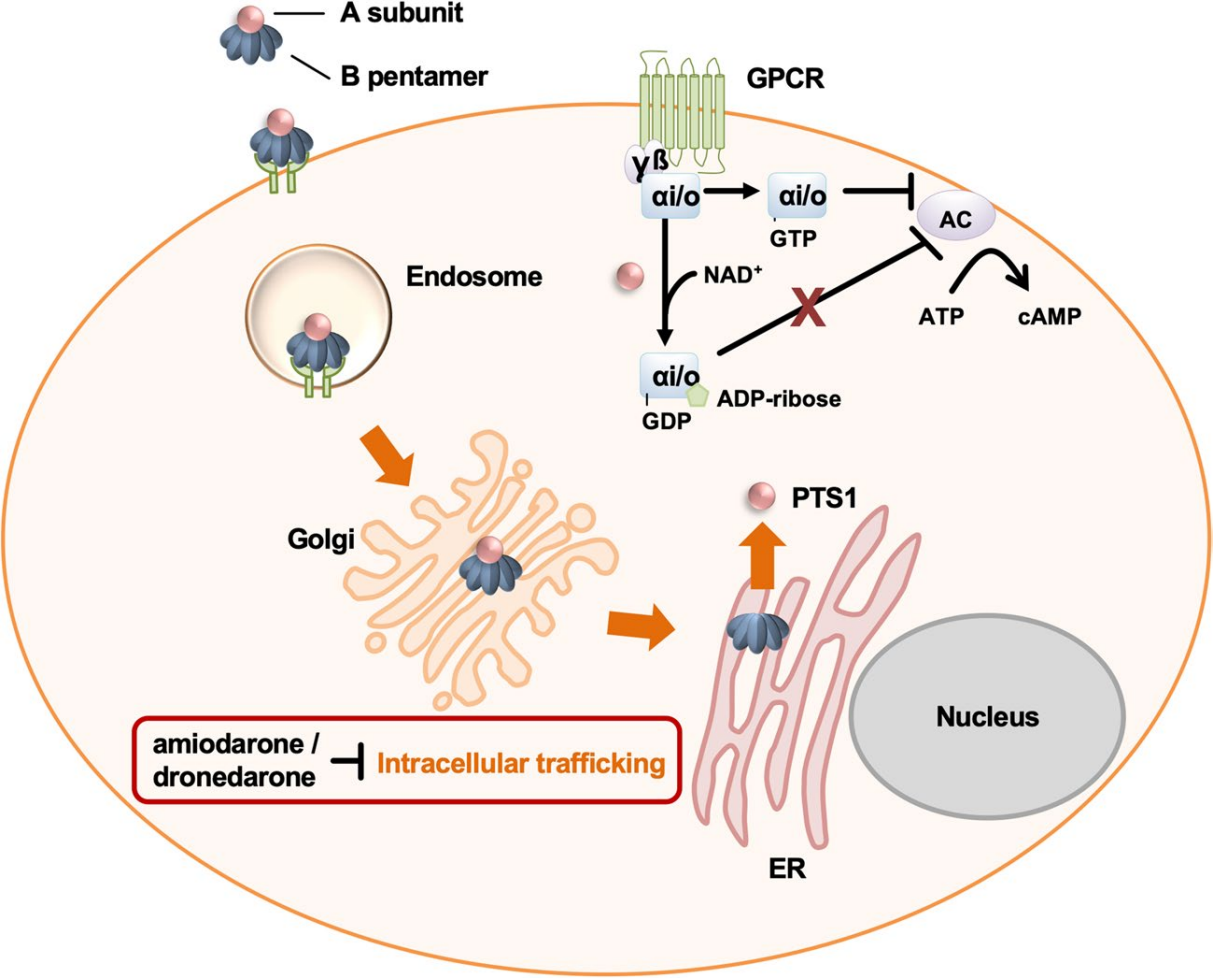
Proposed inhibition mechanism of PT by amiodarone/dronedarone

Our study adds to this body of knowledge by revealing that amiodarone interferes with the intracellular trafficking of the long-trip toxin PT (Fig. 6). PT binding to cells and enzyme activity in vitro were not affected by amiodarone but less PTS1 was detected in cells upon incubation with amiodarone. This suggests that amiodarone also interferes with intracellular trafficking of PT. Although CT and PT are closely related and are both long trip toxins, differences in their uptake mechanism have been described before. For example, they use different cell surface receptors and translocation to the cytosol is assisted by partially distinct host cell chaperones (Armstrong et al. 1988; Wernick et al. 2010; Burress et al. 2019; Kellner et al. 2019, 2021; Ernst et al. 2021).

**Fig. 7:**
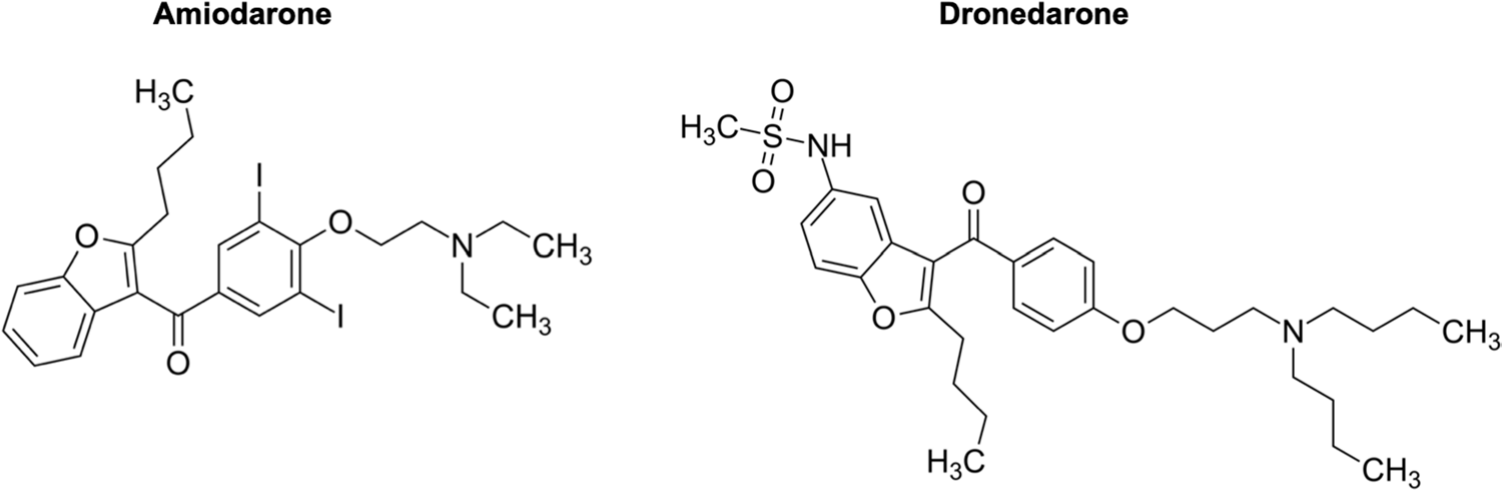
Structures of amiodarone and dronedarone. Dronedarone lacks iodine atoms present in amiodarone, which reduces its thyroid toxicity

Steady state plasma concentrations of amiodarone were measured between ca. 0.6 and 19 µM (0.4 to 11.99 µg/ml) and fall within a similar range with concentrations used in our study (Latini et al. 1984). However, amiodarone can exhibit severe side effects due to extracardiac toxicity. Prevalence in the first year of treatment is ca. 15% and can reach up to 50% in long-term use (Raeder et al. 1985; Goldschlager et al. 2007; Barra et al. 2022). In case of treating pertussis or other diseases caused by bacterial toxins such as TcdA/TcdB, the duration of treatment can be expected to be shorter, therefore possibly reducing the risk of these adverse effects. Amiodarone was also shown to directly activate PT-sensitive G proteins (Hagelüken et al. 1995). However, dronedarone, a derivative of amiodarone, exhibits protective effects similar to amiodarone against PT intoxication and attenuates PT-induced effects on cAMP signaling in a cell-based bioassay. Dronedarone was developed as a derivative of amiodarone to improve the safety profile. It lacks the iodine moiety present in amiodarone and has therefore fewer thyroid-related side effects (Fig. 7). When dronedarone is administered repeatedly at a dose of 400 mg twice daily (orally), it exhibits a mean accumulation ratio ranging from 2.6 to 4.5. The maximum steady-state concentration is observed to be between 84 and 167 ng/mL, corresponding to 0.15 to 0.3 µM (Dorian 2010; Iram et al. 2016). Inhibition of PT-intoxication started to show at concentrations 0.03 to 0.5 µM, and became more pronounced at 5 to 15 µM depending on the readout. Dronedarone also has a shorter half-life leading to potentially faster clearance and reduced risk of accumulating toxic levels (Yalta et al. 2009; Khan et al. 2017).

In conclusion, our study provides evidence supporting the inhibitory effects of amiodarone and dronedarone on PT-induced cellular intoxication. These findings underscore the potential of repurposing existing drugs for the treatment of infectious diseases and pave the way for further research into novel therapeutic strategies against pertussis. By leveraging the pharmacological properties of existing drugs, such as amiodarone and dronedarone, we may uncover novel strategies for combating pertussis and other infectious diseases while expanding our understanding of drug repurposing and cellular signaling pathways.

## Data Availability

All data generated or analyzed during this study are included in this published article.

## References

[CR1] Allen LB, Genaro-Mattos TC, Anderson A et al (2020) Amiodarone alters cholesterol biosynthesis through tissue-dependent inhibition of emopamil binding protein and dehydrocholesterol reductase 24. ACS Chem Neurosci 11:1413–1423. 10.1021/acschemneuro.0c00042 32286791 10.1021/acschemneuro.0c00042 PMC7970401

[CR2] Althouse BM, Scarpino SV (2015) Asymptomatic transmission and the resurgence of Bordetella pertussis. BMC Med 13:146. 10.1186/s12916-015-0382-8 26103968 10.1186/s12916-015-0382-8 PMC4482312

[CR3] Armstrong GD, Howard LA, Peppler MS (1988) Use of glycosyltransferases to restore pertussis toxin receptor activity to asialoagalactofetuin. J Biol Chem 263:8677–8684 2454226

[CR4] Ashok Y, Miettinen M, de Oliveira DKH et al (2020) Discovery of compounds inhibiting the ADP-ribosyltransferase activity of pertussis toxin. ACS Infect Dis 6:588–602. 10.1021/acsinfecdis.9b00412 31899865 10.1021/acsinfecdis.9b00412

[CR5] Banerjee T, Cilenti L, Taylor M et al (2016) Thermal unfolding of the pertussis toxin s1 subunit facilitates toxin translocation to the cytosol by the mechanism of endoplasmic reticulum-associated degradation. Infect Immun 84:3388–3398. 10.1128/IAI.00732-16 27647866 10.1128/IAI.00732-16 PMC5116717

[CR6] Barra S, Primo J, Gonçalves H et al (2022) Is amiodarone still a reasonable therapeutic option for rhythm control in atrial fibrillation? Rev Port Cardiol 41:783–789. 10.1016/j.repc.2021.03.019 36066275 10.1016/j.repc.2021.03.019

[CR7] Barsi S, Papp H, Valdeolivas A et al (2022) Computational drug repurposing against SARS-CoV-2 reveals plasma membrane cholesterol depletion as key factor of antiviral drug activity. PLoS Comput Biol 18:e1010021. 10.1371/journal.pcbi.1010021 35404937 10.1371/journal.pcbi.1010021 PMC9022874

[CR8] Bokoch GM, Katada T, Northup JK et al (1983) Identification of the predominant substrate for ADP-ribosylation by islet activating protein. J Biol Chem 258:2072–2075 6296122

[CR9] Burns DL, Manclark CR (1986) Adenine nucleotides promote dissociation of pertussis toxin subunits. J Biol Chem 261:4324–4327 3005329

[CR10] Burress H, Kellner A, Guyette J et al (2019) HSC70 and HSP90 chaperones perform complementary roles in translocation of the cholera toxin A1 subunit from the endoplasmic reticulum to the cytosol. J Biol Chem. 10.1074/jbc.RA119.008568 31221799 10.1074/jbc.RA119.008568 PMC6690686

[CR11] Carbonetti NH (2015) Contribution of pertussis toxin to the pathogenesis of pertussis disease. Pathog Dis 73:ftv073. 10.1093/femspd/ftv073 10.1093/femspd/ftv073 PMC4626579 26394801

[CR12] Carbonetti NH (2016) Pertussis leukocytosis: mechanisms, clinical relevance and treatment. Pathog Dis 74. 10.1093/femspd/ftw087 10.1093/femspd/ftw087 PMC5761200 27609461

[CR13] Dorian P (2010) Clinical pharmacology of dronedarone: implications for the therapy of atrial fibrillation. J Cardiovasc Pharmacol Ther 15:15S-18S. 10.1177/1074248410367792 20472816 10.1177/1074248410367792

[CR14] el Bayâ A, Linnemann R, von Olleschik-Elbheim L et al (1997) Endocytosis and retrograde transport of pertussis toxin to the Golgi complex as a prerequisite for cellular intoxication. Eur J Cell Biol 73:40–48 9174670

[CR15] Ernst K (2022) Novel strategies to inhibit pertussis toxin. Toxins (basel) 14:187. 10.3390/toxins14030187 35324684 10.3390/toxins14030187 PMC8951090

[CR16] Ernst K, Eberhardt N, Mittler A-K et al (2018) Pharmacological cyclophilin inhibitors prevent intoxication of mammalian cells with Bordetella pertussis toxin. Toxins 10:181. 10.3390/toxins10050181 29723951 10.3390/toxins10050181 PMC5983237

[CR17] Ernst K, Mittler A-K, Winkelmann V et al (2021) Pharmacological targeting of host chaperones protects from pertussis toxin in vitro and in vivo. Sci Rep 11:5429. 10.1038/s41598-021-84817-2 33686161 10.1038/s41598-021-84817-2 PMC7940712

[CR18] Esposito S, Stefanelli P, Fry NK et al (2019) Pertussis prevention: reasons for resurgence, and differences in the current acellular pertussis vaccines. Front Immunol 10:1344. 10.3389/fimmu.2019.01344 31333640 10.3389/fimmu.2019.01344 PMC6616129

[CR19] Ghovanloo M-R, Abdelsayed M, Ruben PC (2016) Effects of amiodarone and n-desethylamiodarone on cardiac voltage-gated sodium channels. Front Pharmacol 7:39. 10.3389/fphar.2016.00039 26973526 10.3389/fphar.2016.00039 PMC4771766

[CR20] Giesemann T, Jank T, Gerhard R et al (2006) Cholesterol-dependent pore formation of Clostridium difficile toxin A *. J Biol Chem 281:10808–10815. 10.1074/jbc.M512720200 16513641 10.1074/jbc.M512720200

[CR21] Goldschlager N, Epstein AE, Naccarelli GV et al (2007) A practical guide for clinicians who treat patients with amiodarone: 2007. Heart Rhythm 4:1250–1259. 10.1016/j.hrthm.2007.07.020 17765636 10.1016/j.hrthm.2007.07.020

[CR22] Hagelüken A, Nürnberg B, Harhammer R et al (1995) The class III antiarrhythmic drug amiodarone directly activates pertussis toxin-sensitive G proteins. Mol Pharmacol 47:234–240 7870030

[CR23] Hausman SZ, Burns DL (1993) Binding of pertussis toxin to lipid vesicles containing glycolipids. Infect Immun 61:335–337 8418057 10.1128/iai.61.1.335-337.1993 PMC302725

[CR24] Hazes B, Read RJ (1997) Accumulating evidence suggests that several AB-toxins subvert the endoplasmic reticulum-associated protein degradation pathway to enter target cells. Biochemistry 36:11051–11054. 10.1021/bi971383p 9333321 10.1021/bi971383p

[CR25] Hazes B, Boodhoo A, Cockle SA, Read RJ (1996) Crystal structure of the pertussis toxin-ATP complex: a molecular sensor. J Mol Biol 258:661–671. 10.1006/jmbi.1996.0277 8637000 10.1006/jmbi.1996.0277

[CR26] Iram F, Ali S, Ahmad A et al (2016) A review on dronedarone: pharmacological, pharmacodynamic and pharmacokinetic profile. Journal of Acute Disease 5:102–108. 10.1016/j.joad.2015.10.002

[CR27] Jia J, Zoeschg M, Barth H et al (2024) The chaperonin TRiC/CCT inhibitor HSF1A protects cells from intoxication with pertussis toxin. Toxins (basel) 16:36. 10.3390/toxins16010036 38251252 10.3390/toxins16010036 PMC10819386

[CR28] Katada T, Ui M (1982) Direct modification of the membrane adenylate cyclase system by islet-activating protein due to ADP-ribosylation of a membrane protein. Proc Natl Acad Sci U S A 79:3129–3133 6954463 10.1073/pnas.79.10.3129 PMC346367

[CR29] Kellner A, Cherubin P, Harper JK, Teter K (2021) Proline isomerization as a key determinant for Hsp90-toxin interactions. Front Cell Infect Microbiol 11:771653. 10.3389/fcimb.2021.771653 34746036 10.3389/fcimb.2021.771653 PMC8569296

[CR30] Kellner A, Taylor M, Banerjee T, et al (2019) A binding motif for Hsp90 in the A chains of ADP-ribosylating toxins that move from the endoplasmic reticulum to the cytosol. Cell Microbiol e13074. 10.1111/cmi.13074 10.1111/cmi.13074 PMC6744307 31231933

[CR31] Khan MH, Rochlani Y, Aronow WS (2017) Efficacy and safety of dronedarone in the treatment of patients with atrial fibrillation. Expert Opin Drug Saf 16:1407–1412. 10.1080/14740338.2017.1387246 28960089 10.1080/14740338.2017.1387246

[CR32] Kilgore PE, Salim AM, Zervos MJ, Schmitt H-J (2016) Pertussis: microbiology, disease, treatment, and prevention. Clin Microbiol Rev 29:449–486. 10.1128/CMR.00083-15 27029594 10.1128/CMR.00083-15 PMC4861987

[CR33] Kling C, Pulliainen AT, Barth H, Ernst K (2021) Human peptides α-defensin-1 and -5 inhibit pertussis toxin. Toxins (basel) 13:480. 10.3390/toxins13070480 34357952 10.3390/toxins13070480 PMC8310310

[CR34] Kodama I, Kamiya K, Toyama J (1997) Cellular electropharmacology of amiodarone. Cardiovasc Res 35:13–29. 10.1016/s0008-6363(97)00114-4 9302343 10.1016/s0008-6363(97)00114-4

[CR35] Latini R, Tognoni G, Kates RE (1984) Clinical pharmacokinetics of amiodarone. Clin Pharmacokinet 9:136–156. 10.2165/00003088-198409020-00002 6370540 10.2165/00003088-198409020-00002

[CR36] Locht C, Antoine R (2021) The History of Pertussis Toxin Toxins (basel) 13:623. 10.3390/toxins13090623 34564627 10.3390/toxins13090623 PMC8472871

[CR37] Locht C, Coutte L, Mielcarek N (2011) The ins and outs of pertussis toxin. FEBS J 278:4668–4682. 10.1111/j.1742-4658.2011.08237.x 21740523 10.1111/j.1742-4658.2011.08237.x

[CR38] Mattoo S, Cherry JD (2005) Molecular pathogenesis, epidemiology, and clinical manifestations of respiratory infections due to Bordetella pertussis and other Bordetella subspecies. Clin Microbiol Rev 18:326–382. 10.1128/CMR.18.2.326-382.2005 15831828 10.1128/CMR.18.2.326-382.2005 PMC1082800

[CR39] Matylitsky J, Krieg A, Schumacher J, Borho J, Barth H, Papatheodorou P (2024) Inhibition of Clostridioides difficile toxins TcdA and TcdB by the amiodarone derivative dronedarone. Naunyn Schmiedebergs Arch Pharmacol. 10.1007/s00210-024-03248-8 10.1007/s00210-024-03248-8 PMC11582217 38935126

[CR40] Nattel S, Singh BN (1999) Evolution, mechanisms, and classification of antiarrhythmic drugs: focus on class III actions. Am J Cardiol 84:11R-19R. 10.1016/s0002-9149(99)00697-9 10568655 10.1016/s0002-9149(99)00697-9

[CR41] Paddock CD, Sanden GN, Cherry JD et al (2008) Pathology and pathogenesis of fatal bordetella pertussis infection in infants. Clin Infect Dis 47:328–338. 10.1086/589753 18558873 10.1086/589753

[CR42] Pande AH, Moe D, Jamnadas M et al (2006) The pertussis toxin S1 subunit is a thermally unstable protein susceptible to degradation by the 20S proteasome. Biochemistry 45:13734–13740. 10.1021/bi061175+ 17105192 10.1021/bi061175+ PMC2518456

[CR43] Papatheodorou P, Song S, López-Ureña D et al (2019) Cytotoxicity of Clostridium difficile toxins A and B requires an active and functional SREBP-2 pathway. FASEB J 33:4883–4892. 10.1096/fj.201801440R 30592645 10.1096/fj.201801440R

[CR44] Papatheodorou P, Kindig S, Badilla-Lobo A, et al (2021) The compound U18666A inhibits the intoxication of cells by clostridioides difficile toxins TcdA and TcdB. Frontiers in Microbiology 12:10.3389/fmicb.2021.784856 PMC8667575 34912322

[CR45] Paramonov VM, Sahlgren C, Rivero-Müller A, Pulliainen AT (2020) iGIST-A kinetic bioassay for pertussis toxin based on its effect on inhibitory GPCR signaling. ACS Sens 5:3438–3448. 10.1021/acssensors.0c01340 33147407 10.1021/acssensors.0c01340 PMC7706119

[CR46] Piccoli E, Nadai M, Caretta CM et al (2011) Amiodarone impairs trafficking through late endosomes inducing a Niemann-Pick C-like phenotype. Biochem Pharmacol 82:1234–1249. 10.1016/j.bcp.2011.07.090 21878321 10.1016/j.bcp.2011.07.090 PMC7092840

[CR47] Pittman M (1984) The concept of pertussis as a toxin-mediated disease. Pediatr Infect Dis 3:467–486 6093069 10.1097/00006454-198409000-00019

[CR48] Plaut RD, Carbonetti NH (2008) Retrograde transport of pertussis toxin in the mammalian cell. Cell Microbiol 10:1130–1139. 10.1111/j.1462-5822.2007.01115.x 18201245 10.1111/j.1462-5822.2007.01115.x

[CR49] Plaut RD, Scanlon KM, Taylor M, et al (2016) Intracellular disassembly and activity of pertussis toxin require interaction with ATP. Pathog Dis 74:. 10.1093/femspd/ftw065 10.1093/femspd/ftw065 PMC5761283 27369899

[CR50] Raeder EA, Podrid PJ, Lown B (1985) Side effects and complications of amiodarone therapy. Am Heart J 109:975–983. 10.1016/0002-8703(85)90238-8 3158188 10.1016/0002-8703(85)90238-8

[CR51] Sakari M, Tran MT, Rossjohn J et al (2022) Crystal structures of pertussis toxin with NAD+ and analogs provide structural insights into the mechanism of its cytosolic ADP-ribosylation activity. J Biol Chem 298:101892. 10.1016/j.jbc.2022.101892 35378130 10.1016/j.jbc.2022.101892 PMC9079181

[CR52] Sanchez AM, Thomas D, Gillespie EJ et al (2007) Amiodarone and bepridil inhibit anthrax toxin entry into host cells. Antimicrob Agents Chemother 51:2403–2411. 10.1128/AAC.01184-06 17485504 10.1128/AAC.01184-06 PMC1913235

[CR53] Scanlon K, Skerry C, Carbonetti N (2019) Association of pertussis toxin with severe pertussis disease. Toxins (Basel) 11:. 10.3390/toxins11070373 10.3390/toxins11070373 PMC6669598 31252532

[CR54] Schumacher J, Nienhaus A, Heber S et al (2023) Exploring the inhibitory potential of the antiarrhythmic drug amiodarone against Clostridioides difficile toxins TcdA and TcdB. Gut Microbes 15:2256695. 10.1080/19490976.2023.2256695 37749884 10.1080/19490976.2023.2256695 PMC10524773

[CR55] Simonen P, Li S, Chua NK et al (2020) Amiodarone disrupts cholesterol biosynthesis pathway and causes accumulation of circulating desmosterol by inhibiting 24-dehydrocholesterol reductase. J Intern Med 288:560–569. 10.1111/joim.13095 32415867 10.1111/joim.13095

[CR56] Singh BN, Vaughan Williams EM (1970) The effect of amiodarone, a new anti-anginal drug, on cardiac muscle. Br J Pharmacol 39:657–667. 10.1111/j.1476-5381.1970.tb09891.x 5485142 10.1111/j.1476-5381.1970.tb09891.x PMC1702721

[CR57] Stein PE, Boodhoo A, Armstrong GD et al (1994) The crystal structure of pertussis toxin. Structure 2:45–57 8075982 10.1016/s0969-2126(00)00007-1

[CR58] Tamura M, Nogimori K, Murai S et al (1982) Subunit structure of islet-activating protein, pertussis toxin, in conformity with the A-B model. Biochemistry 21:5516–5522 6293544 10.1021/bi00265a021

[CR59] Weiss AA, Johnson FD, Burns DL (1993) Molecular characterization of an operon required for pertussis toxin secretion. Proc Natl Acad Sci USA 90:2970–2974 8464913 10.1073/pnas.90.7.2970 PMC46218

[CR60] Wernick NLB, Chinnapen DJ-F, Cho JA, Lencer WI (2010) Cholera toxin: an intracellular journey into the cytosol by way of the endoplasmic reticulum. Toxins (basel) 2:310–325. 10.3390/toxins2030310 22069586 10.3390/toxins2030310 PMC3153193

[CR61] Witvliet MH, Burns DL, Brennan MJ et al (1989) Binding of pertussis toxin to eucaryotic cells and glycoproteins. Infect Immun 57:3324–3330. 10.1128/IAI.57.11.3324-3330.1989 2478471 10.1128/iai.57.11.3324-3330.1989 PMC259811

[CR62] Worthington ZEV, Carbonetti NH (2007) Evading the proteasome: absence of lysine residues contributes to pertussis toxin activity by evasion of proteasome degradation. Infect Immun 75:2946–2953. 10.1128/IAI.02011-06 17420233 10.1128/IAI.02011-06 PMC1932868

[CR63] Wu L, Rajamani S, Shryock JC et al (2008) Augmentation of late sodium current unmasks the proarrhythmic effects of amiodarone. Cardiovasc Res 77:481–488. 10.1093/cvr/cvm069 18006430 10.1093/cvr/cvm069 PMC2365898

[CR64] Yalta K, Turgut OO, Yilmaz MB et al (2009) Dronedarone: a promising alternative for the management of atrial fibrillation. Cardiovasc Drugs Ther 23:385–393. 10.1007/s10557-009-6189-0 19669399 10.1007/s10557-009-6189-0

[CR65] Yeung KHT, Duclos P, Nelson EAS, Hutubessy RCW (2017) An update of the global burden of pertussis in children younger than 5 years: a modelling study. Lancet Infect Dis 17:974–980. 10.1016/S1473-3099(17)30390-0 28623146 10.1016/S1473-3099(17)30390-0

